# Local pamidronate influences fracture healing in a rodent femur fracture model: an experimental study

**DOI:** 10.1186/s12891-016-1113-9

**Published:** 2016-06-09

**Authors:** Leif Menzdorf, Matthias Weuster, Tim Klüter, Stefan Brüggemann, Peter Behrendt, Stefanie Fitchen-Oestern, Deike Varoga, Andreas Seekamp, Nicolai Purcz, Claus C Glueer, Thomas Pufe, Sebastian Lippross

**Affiliations:** Department of Trauma Surgery, University Medical Center of Schleswig-Holstein, Campus Kiel, Kiel, Germany; Department of Oral and Maxillofacial Surgery, University Medical Center of Schleswig-Holstein, Campus Kiel, Kiel, Germany; Section of Biomedical Imaging, Department of Radiology and Neuroradiology, University Medical Center of Schleswig-Holstein, Campus Kiel, Kiel, Germany; Department of Anatomy and Cell Biology, RWTH Aachen University, Aachen, Germany

## Abstract

**Background:**

Bisphosphonates are a main component in the therapy of osteoporosis and other bone resorptive diseases. Previous studies have shown a positive effect of systemically applied bisphosphonates on fracture healing. Nevertheless high doses are related to side effects like osteonecrosis of the jaw, nephrotoxis and gastrointestinal symptoms. In this study we investigated the effect of locally applied pamidronate on fracture healing.

**Methods:**

In a rodent model a simple femur fracture was set in female Wistar rats. We performed intramedullary fixation of the fracture and placed a collagen matrix around the fracture area. One group was treated with pamidronate, the other group with placebo via the matrix.

To investigate the volume and quality of the callus we used micro-CT (μCT) and histology after 14 and 28 days.

**Results:**

Our results show a positive influence of local applied pamidronate on callus volume. After 14 days an insignificant increase of callus volume in the treated animals was seen. 28 days after trauma the increase of callus volume in the treatment group was significantly higher in comparison to the control group. Osteonecrosis was not seen.

**Conclusions:**

Locally applied bisphosphonates increase the callus volume in fracture healing.

## Background

Osteoporosis is a major health problem of the elderly western society and gains economical importance [1, 2] besides its obvious impact on clinical practice. The risk of fractures is directly related to bone density [3]. In addition the incidence of falls and fall-related injuries rises with age [4]. Therefore fracture fixation in osteopenic bone has become a major task in modern orthopaedic surgery. Postoperative implant loosening is often observed, and ‘cutting out’ of implants in the femoral head is a common complication. In the elderly a rapid mobilisation after surgery is necessary to reduce the incidence of complications like pneumonia and venous thromboembolism as well as mental confusion.

Bisphosphonates are a main component of osteoporosis therapy and other diseases involving increased bone resorption. Osteoporosis is initially treated with oral calcium, vitamin D and Bisphosphonates [5]. Bisphosphonates reduce the activity of osteoclasts by binding hydroxyapatite crystals and impair the osteoclasts ability to resorb bone [6]. In the resorption lacuna with its exposed calcium ions on bone surface a high dose of the bisphosphonates accumulates. The agent is then resorbed by the active osteoclast. On a cellular level bisphosphonates inhibit steroid synthesis by inhibiting the farnesyl diphosphate synthase (FPPS) in the mevalonate pathway. Thereby the synthesis of essential lipid sidechains of membrane binding proteins is impaired. In absence of lipid sidechains it is impossible for proteins to bind to the cell membrane and they are functionless [7–9]. Subsequently the activity of the osteoblasts overweighs and bone resorption is reduced [10]. An emerging concept in modern trauma surgery is therefore to develop biological augmentation techniques in order to promote fracture healing.

Previous studies have shown a positive effect of bisphosphonates on fracture healing [11]. In most of these studies bisphosphonates were given systemically. Based on the poor bioavailability of bisphosphonates high doses were necessary. These can cause side effects like osteonecrosis of the jaw, nephrotoxicity, gastrointestinal symptoms, etc. [12]. Wermelin et al. have studied the local effect of bisphosphonates on implant fixation [13]. The authors investigated the fixation of bisphosphonate-coated screws in rat tibias demonstrating improved screw pull out resistance when bisphosphonates were applied.

Greiner et al. [14] investigated locally applied zoledronic acid via coated implants in fractured rodent tibiae. Bending tests and review of plain radiographs revealed improved mechanical stability of the bones of animals treated with zoledronic acid but the results were not supported by histological or high-resolution radiological studies. Therefore we used μCT to image callus formation and callus volume. Histology was performed to investigate cellular processes.

Our rationale was to make use of the positive effects of bisphosphonates in fracture healing while reducing the side effects of systemically application by local application.

## Methods

### Animals

44 female Wistar-rats (mean weight 200 ± 10) were obtained from the local service unit of the author’s institution. The animals were kept one per cage with free access to water and maintenance diet. The rodents were allowed free movement about their cages. The lighting was maintained on a 12-hour light-dark cycle.

After surgery the animals received analgesia (100 mg/kg Metamizol sodium (Novalgin®, Hoechst, Unterschleissheim, Germany) with the drinking water. The animals were sacrificed on day 14 or 28 after the surgical procedure.

### Surgical procedure

The animals were randomized in two groups. The first group was treated with pamidronate (Pamifos® - Medac GmbH, Wedel) 0.6 mg/kg, the other group was treated with placebo (NaCl 0.9 % Fresenius Kabi AG, Bad Homburg). Anaesthesia was applied (Fentanyl 0.005 mg/kg, Midazolam 2.0 mg/kg und Medetomidin 0.15 mg/kg) intraperitoneally. First the intramedullary fixation through a median parapatellar insertion at the knee was performed as described by Gabet et al. [15]. A cannula was drilled into the fossa intercondylaris and advanced into the femur. Then the wound was closed and a standard trauma was applied to the right femur as described by Bonnarens [16]. Afterwards through a second surgical approach on the lateral femur, a sub vastus approach, the collagen matrix (soaked with pamidronate or placebo) was laid around the fracture region and fixed with a suture. The wound was closed again and anaesthesia was antagonized by Naloxon 0.12 mg/kg, Flumazenil 0.2 mg/kg and Atipamezol 0.75 mg/kg.

### Micro CT analysis

The bones were scanned in a μCT Scanner (vivaCT 40, Scanco Medical AG, Bassersdorf, Switzerland) with 70 kVp, 114 mA and 1300 ms integration time at 10.5 μm resolution in 1000 slices of the fracture region as described before [17]. During scanning, the femoral bones were placed in phosphate buffered saline. For quantitative analysis of the bone formation the μCT Evaluation Program Version 6.5-1 (Sanco Medical, Bassersdorf, Switzerland) was used to obtain the volume of interest. Based on histogram of attenuation distribution, tissue was segmented into highly mineralised tissue (270 – 400) and low mineralised tissue (180 – 269) in per mille of maximal image grey value [15].

### Preparation of specimens and histologic assessment

After the μCT scan bone histology was performed to investigate the micro-architecture of the callus.

For calcified histology the bones were fixed in 10 % neutral buffered formalin for 14 days at room temperature, dehydrated with ascending concentrations of ethanol and embedded in methylmethacrylate for 5 days at 4 °C.

Afterwards the tissue was soaked in methyl methacrylat monomer, nonpylphenyl-polyethyleneglycol acetate and azoisobutyronitrile (all from Sigma-Aldrich, St. Louis, USA). The blocks were released from the glass vials and sections of 40 μm were sawed and grinded using an EXAKT diamond saw system (Exakt, Norderstedt, Germany). The samples were lubricated with 0.1 % formic acid for 2 minutes and washed with water afterwards. The sections were submerged in 20 % methanol for one hour and stained with toluidine blue or haematoxylin and eosin.

### Statistical analysis

We used a standard statistical program (GraphPad Software Inc., La Jolla, CA USA) to perform a Kolmogorov-Smirnov normality test. Statistical significance was evaluated using the Mann-Whitney test. Volumes of were illustrated with box and whisker plots.

## Results

All animals recovered quickly after surgery and mobilized fully within one day. No signs of pain or dysfunction in physical motion were recognized after one day. Five animals died during anaesthesia. No animal had to be excluded because of implant dislocation or soft tissue infection. Six rodents were excluded because of multifragmentary fractures.

### Histology

Callus formation and fracture healing were investigated by standard calcified bone histology. With this method we were able to illustrate the cellular and mineralised components of the callus and the new bone formation.

**Fig. 1:**
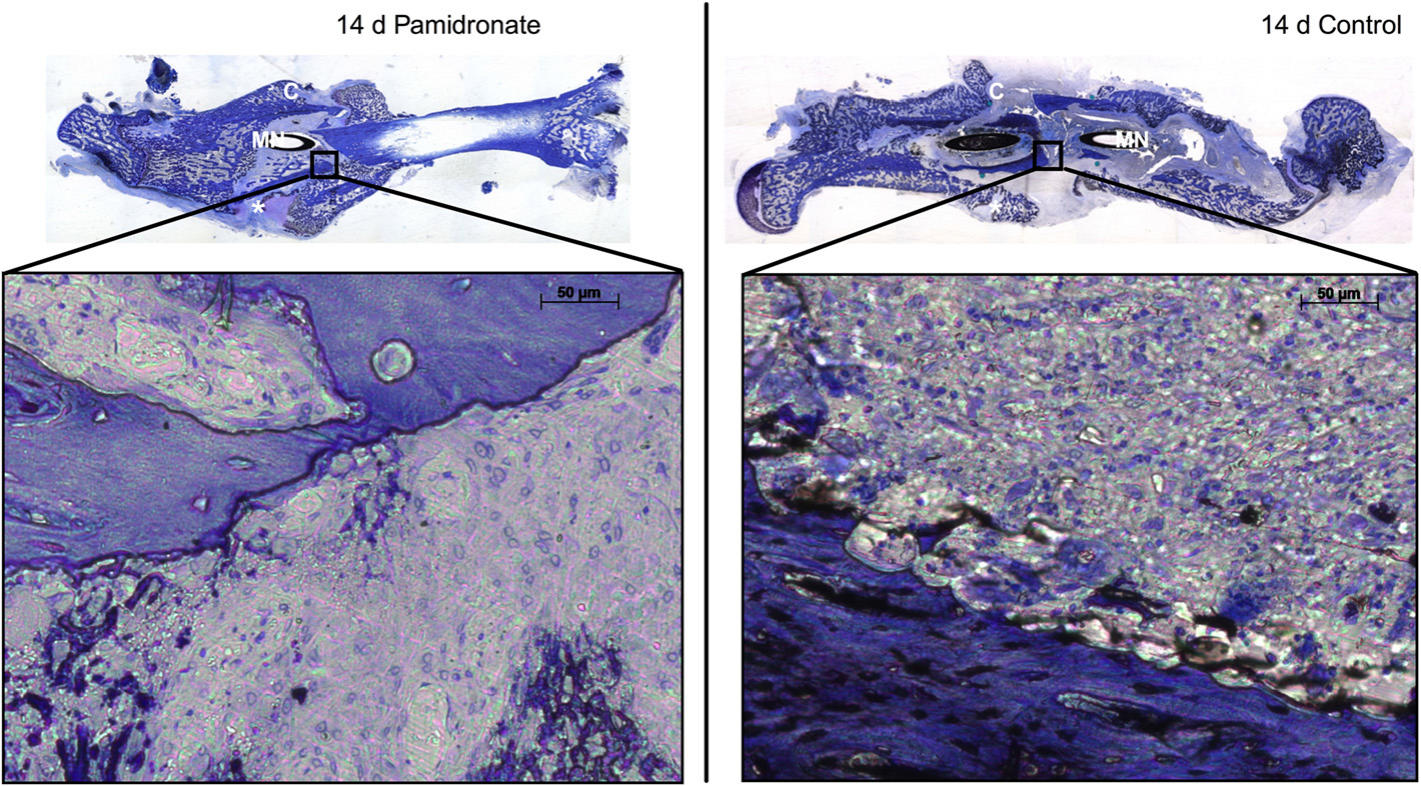
Photomicrography of fracture callus from rat femur. Panoramic views and high magnification photomicrography of fracture healing and callus formation was illustrated using calcified bone histology technique with toluidine blue staining. The magnification points up the remodelling zone within the fracture gap. 14 days after fracture the fracture gap (*) and a sufficient callus (C) is noticeable. (MN) Marknail; (*) fracture gap; (C) callus

14 days after trauma the fracture gap was still visible. Tolouidine blue staining showed the first connective tissue between the bone ends (Fig. 1).

**Fig. 2:**
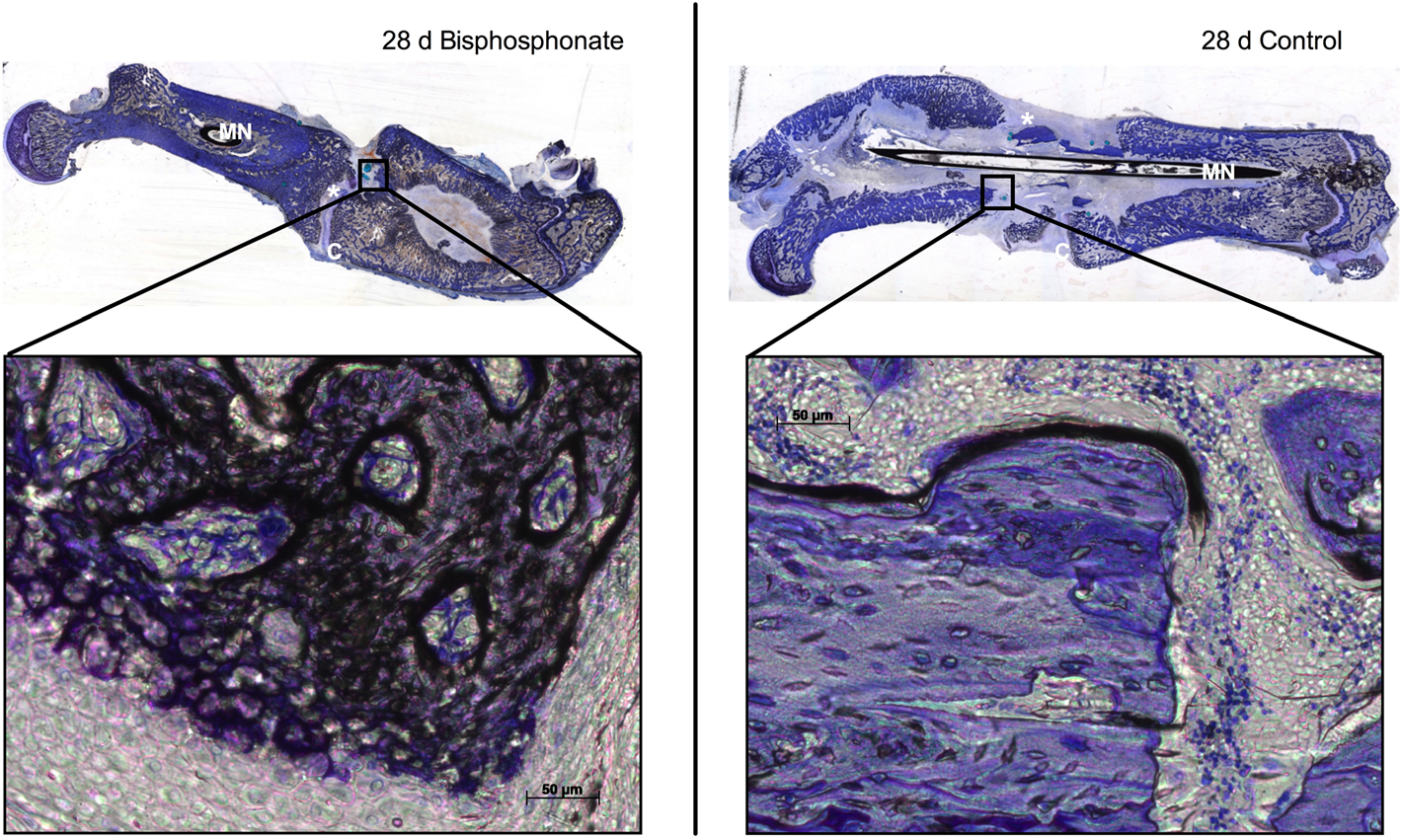
Photomicrography of fracture callus from rat femur. Panoramic views and high magnification photomicrography of fracture healing and callus formation was illustrated using calcified bone histology technique with toluidine blue staining. The magnification points up the remodelling zone within the fracture gap. 28 days after fracture the fracture gap (*) and callus (C) is noticeable. (MN) Marknail; (*) fracture gap; (C) callus

After 28 days the fracture healing was not complete. There was partial bony bridging of the fracture gap (Fig. 2) and sufficient callus had arisen. No clear differences were determined between the two groups.

### Micro CT scans of the fracture zone

**Fig. 3:**
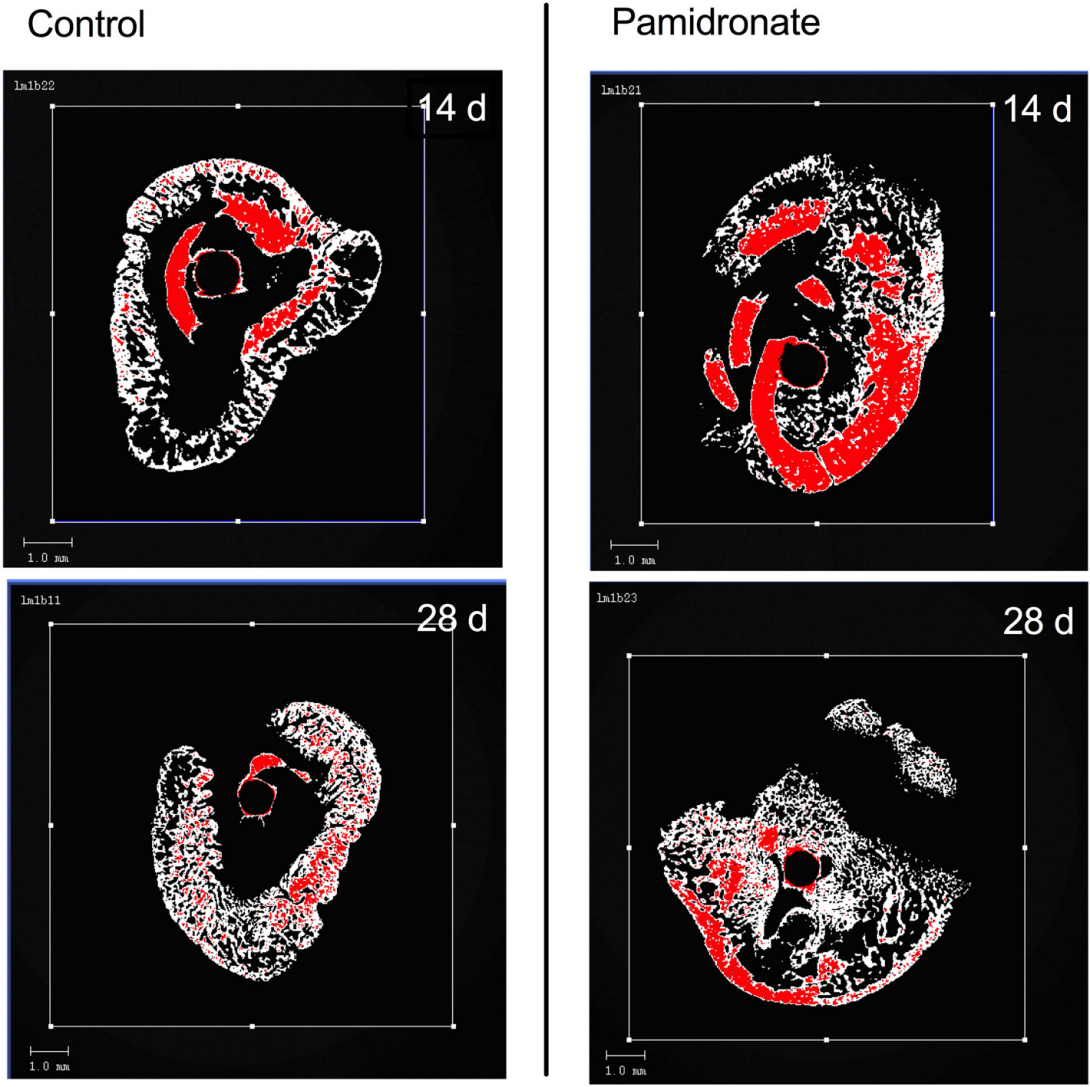
Micro-CT Images of the fracture region. On the right side images of the treated specimens at the different timepoints. On the left images of the control group. Red coloured is the cortical bone (highly mineralised tissue: 270 – 400 per mille of maximal image grey value).

**Fig. 4:**
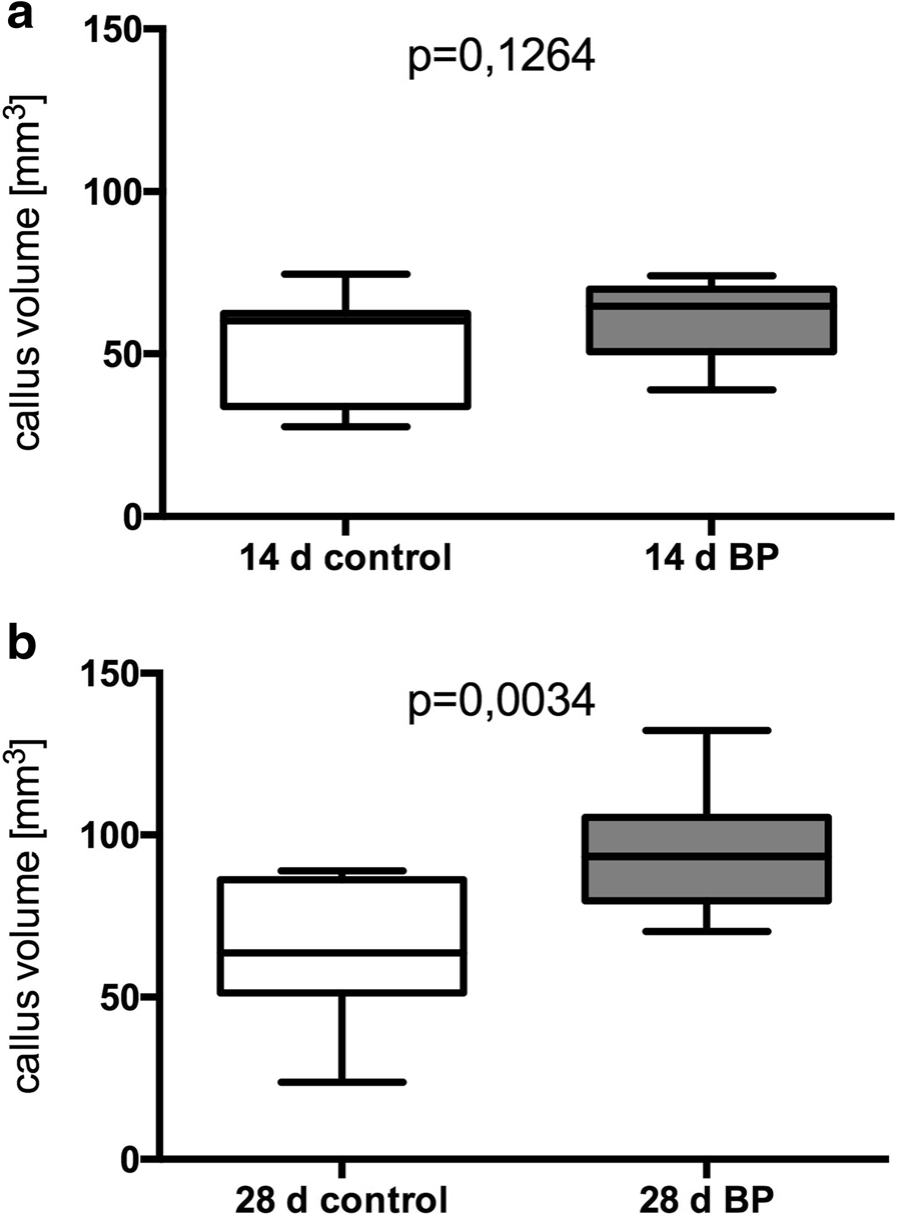
Callus volume measured in μCT. Whiskers from minimum to maximum. **a**: Callus volume 14 days after trauma. On the left untreated group (52.85 mm^3^ ± 16.58 mm^3^; n = 9) and on the right pamidronat treated group (60.94 mm^3^ ± 11.28 mm^3^; n = 12). **b** Callus volume 28 days after trauma. On the left untreated group (63.43 mm^3^ ± 21.49 mm^3^ ; n = 9) and on the right pamidronate treated group (94.03 mm^3^ ± 17.63 mm^3^ ; n = 14).

The callus volume was determined in the fracture region (Fig. 3). After 14 days there was more callus in the animals treated with Bisphosphonates (60.94 mm^3^ ± 11.28 mm^3^; n = 12) than in the control group (52.85 mm^3^ ± 16.58 mm^3^; n = 9). But this increase was statistically not significant (p = 0.1264) (Fig. 4a).

At this time the difference in cortical bone volume was similarly insignificant (BP: 42.01 mm^3^ ± 16.65 mm^3^ ; n = 12; control: 28.14 mm^3^ ± 10.71 mm^3^; n = 9; p = 0.0579).

28 days after trauma the increase of the callus volume in the treated group (94.03 mm^3^ ± 17.63 mm^3^ ; n = 14) was significantly higher (p = 0.0034) in comparison to the control group (63.43 mm^3^ ± 21.49 mm^3^ ; n = 9) (Fig. 4b). 28 days post trauma there was no statistical significant difference (p = 0.4004) in bone volume of treated (60.52 mm^3^ ± 25.08 mm^3^; n = 14) and untreated animals (61.94 mm^3^ ± 20.95 mm^3^ ; n = 9).

## Discussion

In this study we have investigated the effect of local application of bisphosphonate via a bisphosphonate loaded collagen matrix in a well established fracture model.

Previous studies indicate an increase of callus volume in fracture healing under bisphosphonate application [18–20]. In these studies the agent was given systemically. Goodship et al. report that callus volume was increased under treatment with pamidronate in a sheep model. Nyman and colleagues investigated the effect of high doses of subcutaneously injected clodronate on fracture healing in tibia fractures in rats. The results show that there is a higher calcium content in the callus when treated with clodronate [19]. Li et al. demonstrate that significantly larger callus arises when incadronate was injected before and after trauma [21]. Despite all favourable effects described in the literature side effects of the systemic treatment with bisphosphonates do still represent a major obstacle. The group around Villa investigated the bisphosphonate related osteonecrosis of the jaw (BRONJ) in female patients with postmenopausal osteoporosis. With continuous bisphosphonate therapy they report 9 % osteonecrosis of the jaw [22]. Perazella et al. described the nephrotoxicity of intravenous bisphosphonate therapy. The toxicity depends on the dose and the infusion time [23]. Hyldstrup et al. demonstrated in their study the poor bioavailability of oral applied pamidronate. The bioavailability depends on the oral dose and showed high intraindividual variations [24].

Locally applied bisphosphonates therefore offer the distinct advantages of a reduced dose that theoretically causes less side effects and while the local effective dose is increased. So far to our knowledge there is no study investigating the effect of locally applied bisphosphonate on fracture healing.

Skoglund et al. investigated the effect of local applied simvastatin in fracture healing. The group compared the continuous locally applied HMG-CoA reductase inhibitor to daily subcutaneous application. The locally applied simvastatin showed a positive effect on biomechanical properties of the newly formed callus [25].

Our results demonstrate a positive effect on fracture healing of locally applied bisphosphonate. After 14 days the callus volume was increased in the treated group. The difference became statistically significant after 28 days. In keeping with these results previous studies showed increased callus volume after systemically applied bisphosphonates [18, 20].

At no time point the bione volume was significantly increased in either group. After 14 days the bone volume was slightly increased in the treated rodents. After 28 days the increase turned into a slight decrease and the control group showed slightly higher bone volume. A possible explanation for the phenomena is a delayed bone turnover under bisphosphonate therapy as described before [26]. On a cellular level histology does not reveal osteonecrosis, abnormal bone healing or inflammation.

The rodent fracture model we used is an established and commonly used model to investigate fracture healing. It is a cheap and easy to handle model in contrast to larger animals. Nevertheless certain aspects of rodent fracture healing may well be used to investigate basic mechanisms. The limited amount of animals in this study is due to ethical considerations. When all animals included in this study were analysed it seemed inappropriate to further increase the number tested as our results seemed convincing. Statistically there is less power of the cohort.

Limitations of the study presented here comprise lack of mechanical testing, which would be necessary to be investigated in further studies.

## Conclusions

Locally applied bisphosphonates increase the callus volume in fracture healing. There is no increased bone volume under the influence of bisphosphonate. Biomechanical testing will be necessary to investigate the mechanical abilities of the newly formed tissue. With local application lower doses of bisphosphonates may be effective which could lead to a reduction of side effects.

## Abbreviations

BRONJ, bisphosphonate related osteonecrosis of the jaw; FPPS, farnesyl-diphosphate-synthase; HMG-CoA 3-hydroxy-3-methylglutaryl-coenzyme A; μCT, micro computed tomography.

## References

[1] Cole ZA, Dennison EM, Cooper C (2008). Osteoporosis epidemiology update. Curr Rheumatol Rep.

[2] Becker S, Ogon M (2008). Epidemiology of osteoporosis. Balloon Kyphoplasty.

[3] Kanis JA, Melton LJ, Christiansen C, Johnston CC, Khaltaev N (1994). The diagnosis of osteoporosis. J Bone Miner Res.

[4] Towne SD, Smith ML, Yoshikawa A, Ory MG (2015). Geospatial distribution of fall-related hospitalization incidence in Texas. J Safety Res.

[5] Fleisch H (2003). Bisphosphonates in osteoporosis. Eur Spine J.

[6] Riggs BL, Melton LJ (1992). The prevention and treatment of osteoporosis. N Engl J Med.

[7] Reno F, Rizzi M, Invernizzi M, Migliario M, Cisari C (2013). Low doses amino-bisphosphonates stimulate keratinocytes growth inactivating glucocorticoid receptor. Eur J Pharmacol.

[8] Kavanagh KL, Guo K, Dunford JE, Wu X, Knapp S, Ebetino FH, Rogers MJ, Russell RG, Oppermann U (2006). The molecular mechanism of nitrogen-containing bisphosphonates as antiosteoporosis drugs. Proc Natl Acad Sci U S A.

[9] Dunford JE (2010). Molecular targets of the nitrogen containing bisphosphonates: the molecular pharmacology of prenyl synthase inhibition. Curr Pharm Des.

[10] Fleisch H (1991). Bisphosphonates. Pharmacology and use in the treatment of tumour- induced hypercalcaemic and metastatic bone disease. Drugs.

[11] Amanat N, Brown R, Bilston LE, Little DG (2005). A single systemic dose of pamidronate improves bone mineral content and accelerates restoration of strength in a rat model of fracture repair. J Orthop Res.

[12] Elliott SN, McKnight W, Davies NM, MacNaughton WK, Wallace JL (1998). Alendronate induces gastric injury and delays ulcer healing in rodents. Life Sci.

[13] Wermelin K, Suska F, Tengvall P, Thomsen P, Aspenberg P (2008). Stainless steel screws coated with bisphosphonates gave stronger fixation and more surrounding bone. Histomorphometry in rats. Bone.

[14] Greiner SH, Wildemann B, Back DA, Alidoust M, Schwabe P, Haas NP, Schmidmaier G (2008). Local application of zoledronic acid incorporated in a poly(D, L-lactide)-coated implant accelerates fracture healing in rats. Acta Orthop.

[15] Gabet Y, Muller R, Regev E, Sela J, Shteyer A, Salisbury K, Chorev M, Bab I (2004). Osteogenic growth peptide modulates fracture callus structural and mechanical properties. Bone.

[16] Bonnarens F, Einhorn TA (1984). Production of a standard closed fracture in laboratory animal bone. J Orthop Res.

[17] Grongroft I, Heil P, Matthys R, Lezuo P, Tami A, Perren S, Montavon P, Ito K (2009). Fixation compliance in a mouse osteotomy model induces two different processes of bone healing but does not lead to delayed union. J Biomech.

[18] Goodship AE, Walker PC, McNally D, Chambers T, Green JR (1994). Use of a bisphosphonate (pamidronate) to modulate fracture repair in ovine bone. Ann Oncol.

[19] Nyman MT, Paavolainen P, Lindholm TS (1993). Clodronate increases the calcium content in fracture callus. An experimental study in rats. Arch Orthop Trauma Surg.

[20] Li J, Mori S, Kaji Y, Kawanishi J, Akiyama T, Norimatsu H (2000). Concentration of bisphosphonate (incadronate) in callus area and its effects on fracture healing in rats. J Bone Miner Res.

[21] Li C, Mori S, Li J, Kaji Y, Akiyama T, Kawanishi J, Norimatsu H (2001). Long-term effect of incadronate disodium (YM-175) on fracture healing of femoral shaft in growing rats. J Bone Miner Res.

[22] Villa A, Castiglioni S, Peretti A, Omodei M, Ferrieri GB, Abati S (2011). Osteoporosis and bisphosphonate-related osteonecrosis of the jaw bone. ISRN Rheumatol.

[23] Perazella MA, Markowitz GS (2008). Bisphosphonate nephrotoxicity. Kidney Int.

[24] Hyldstrup L, Flesch G, Hauffe SA (1993). Pharmacokinetic evaluation of pamidronate after oral administration: a study on dose proportionality, absolute bioavailability, and effect of repeated administration. Calcif Tissue Int.

[25] Skoglund B, Aspenberg P (2007). Locally applied Simvastatin improves fracture healing in mice. BMC Musculoskelet Disord.

[26] Komatsubara S, Mori S, Mashiba T, Li J, Nonaka K, Kaji Y, Akiyama T, Miyamoto K, Cao Y, Kawanishi J (2004). Suppressed bone turnover by long-term bisphosphonate treatment accumulates microdamage but maintains intrinsic material properties in cortical bone of dog rib. J Bone Miner Res.

